# Quality Indicators for Continuous Monitoring to Improve Maternal and Infant Health in Maternity Departments: A Modified Delphi Survey of an International Multidisciplinary Panel

**DOI:** 10.1371/journal.pone.0060663

**Published:** 2013-04-05

**Authors:** Rym Boulkedid, Olivier Sibony, François Goffinet, Arnaud Fauconnier, Bernard Branger, Corinne Alberti

**Affiliations:** 1 AP-HP, Hôpital Robert Debré, Unité d’Epidémiologie Clinique, Paris, France; 2 Institut National de la Santé et de la Recherche Médicale, CIE 5, Paris, France; 3 AP-HP, Hôpital Robert Debré, Pôle Gynécologie et périnatalité, Hospitalisation Gynécologie-Obstétrique, Paris, France; 4 Univ Paris Diderot, Sorbonne Paris Cité, Paris, France; 5 Institut National de la Santé et de la Recherche Médicale, Unité de Recherche épidémiologique en santé périnatale et santé des femmes et des enfants, U953, UPMC, Paris, France; 6 AP-HP, Hôpital Cochin Broca Hôtel-Dieu, Maternité Port Royal, Paris, France; 7 Univ Paris-Descartes, Sorbonne Paris Cité, Paris, France; 8 CHI Poissy-Saint Germain en Laye, Service de Gynécologie Obstétrique, Poissy, France; 9 Réseau Sécurité Naissance – Naître Ensemble, Pays-de-Loire, Nantes, France; Tehran University of Medical Sciences, Islamic Republic of Iran

## Abstract

**Objective:**

Measuring the quality of inpatient obstetrical care using quality indicators is becoming increasingly important for both patients and healthcare providers. However, there is no consensus about which measures are optimal. We describe a modified Delphi method to identify a set of indicators for continuously monitoring the quality of maternity care by healthcare professionals.

**Methodology and Main Findings:**

An international French-speaking multidisciplinary panel comprising 22 obstetricians-gynaecologists, 12 midwives, and 1 paediatrician assessed potential indicators extracted from a medical literature search, using a two-round Delphi procedure followed by a physical meeting. Each panellist rated each indicator based on validity and feasibility. In the first round, 35 panellists from 5 countries and 20 maternity units evaluated 26 indicators including 15 related to the management of the overall population of pregnant women, 3 to the management of women followed from the first trimester of pregnancy, 2 to the management of low-risk pregnant women, and 6 to the management of neonates. 25 quality indicators were kept for next step. In the second round, 27 (27/35: 77%) panellists selected 17 indicators; the remaining 8 indicators were discussed during a physical meeting. The final set comprised 18 indicators.

**Conclusion:**

A multidisciplinary panel selected indicators that reflect the quality of obstetrical care. This set of indicators could be used to assess and monitor obstetrical care, with the goal of improving the quality of care in maternity units.

## Introduction

Healthcare quality measurement has attracted considerable attention in recent years. Health authorities and healthcare professionals have been using a wide range of tools to promote quality improvement. Over the past decade, the development and implementation of quality indicators (also known as performance indicators or quality measures) has been largely driven by the introduction of computerised administrative and clinical databases and by a decision to make performance data available to the public. [1] In addition, it has been shown that continuously monitoring quality indicators significantly improved the quality of care [2], [3].

Measuring the quality of inpatient obstetrical care is becoming increasingly important for both patients and healthcare providers. Indeed, each obstetrical admission may affect the health of not one, but two individuals. In addition, most women admitted for obstetrical reasons are healthy individuals, in whom the goal is full preservation of health, in particular via careful attention to adverse events caused by management errors [4], [5].

Several validated indicators are suggested for evaluating obstetrical care, but their usefulness is unclear. For example, maternal and perinatal mortality rates are still the main indicators used to assess labour outcomes, both nationally and internationally. However, maternal deaths and severe maternal complications (e.g., bleeding and preeclampsia) have become extremely rare and perinatal mortality and morbidity rates (e.g., cerebral palsy rates) lack sensitivity for assessing the various components of obstetrical care. Continuous monitoring of obstetrical care faces several challenges. More specifically, the available data are often flawed and limited, and adverse outcomes are rare and ill-suited to continuous monitoring. Consequently, studies are needed to identify valid quality indicators for maternity units.

One method used to develop quality indicators is the Delphi technique, which relies directly on the available evidence, complemented with expert opinion when needed. [6], [7] The Delphi technique has been used to develop prescribing indicators, [8] indicators reflecting patient and general practitioner perceptions of chronic illness, [9] performance indicators for emergency medicine, [10] and indicators for cardiovascular disease. [11] A modified version of the Delphi technique has served to develop a set of indicators for measuring the quality of clinical care in emergency departments [12] or monitoring neurodevelopmental outcomes in very low birth weight children [13].

Here, we describe the systematic development of quality indicators for obstetrical care as the first step of a quality improvement programme that is being set up in several maternity units in France with the goal of promoting performance excellence and maintaining a high level of quality of care. A modified Delphi technique carried out by an international panel of French-speaking experts in obstetrical care was used to select a set of quality indicators to be monitored routinely and continuously in maternity units using a CUSUM (CUmulative SUM) control chart [14] and to determine the extent to which these indicators were assessable using the existing population-based administrative dataset. Our aim was to produce relevant and valid indicators that could be routinely monitored to guide the development of quality improvement programs.

## Methods

### Setting

This study was part of a quality improvement project under way in several maternity units in France. The working hypothesis is that continuous quality indicator monitoring using a specific method (Cumulative Sum, CUSUM) [14] will increase awareness of quality issues among maternity-unit healthcare workers and permit the rapid detection of small dips in performance, thereby enabling prompt investigations and corrective measures when necessary. As demonstrated in previous work, CUSUM chart monitoring can lead to quality indicator assessments after every 50 or 100 births depending on the occurrence rate of the event.The first step is to achieve a consensus about which quality indicators should be monitored.

### Delphi Survey

A panel of experts participated in a modified two-round Delphi procedure between September 2009 and December 2010. In a Delphi survey, two or more questionnaire rounds are completed to achieve a consensus among panellists selected based on expertise in the relevant field. The questionnaire items are statements on a given issue developed by either the panellists or the researchers. The panellists rate the statements, and the results of each rating round are communicated to them before the next round. [6] One of the main reasons the Delphi technique is popular is that a large number of individuals in various locations and areas of expertise can be included anonymously, without interacting directly with each other, which prevents the views of a minority from dominating the group [15].

We used a modified Delphi technique in which questionnaire rounds were followed by a physical meeting of the panellists, to enhance the complex decision-making process and to clarify the language used to describe each indicator [16], [17], [18].

### Questionnaire Development

In a previous study, we identified existing quality indicators that could be routinely monitored using a maternity dashboard. [14] In brief, a MEDLINE search was conducted using the key words ‘quality indicators’ and ‘obstetrical care’ or ‘prenatal care’. Furthermore, we searched other sources of data such as government reports, electronic databases, organisation websites, and national and international initiatives. We used the results of the literature search to compile a list of non-duplicate quality indicators for obstetrical care. Our efforts to achieve an optimal balance between evaluating all the main steps of the care process and keeping the list of indicators reasonably short led us to select 26 indicators (Appendix S1). Among them, 15 were related to the management of the overall population of pregnant women, 3 to the management of women followed from the first trimester of pregnancy, 2 to the management of low-risk pregnant women, and 6 to the management of neonates.

For each indicator, we provided the panellists with definitions of the population exhibiting the relevant event (numerator) and of the target population used as the denominator. In some cases, however, we found no universally agreed-on definitions of the numerator and/or denominator in the literature and we therefore suggested that the panellists develop definitions during the physical meeting that ended the modified Delphi procedure.

Each panel member was asked to rate each of the 26 quality indicators based on validity and feasibility. An indicator was considered valid if adequate scientific evidence supported a link between the delivery of care due to presence of the indicator and benefits to the patient and physician. An indicator was considered feasible if the information needed to assess it was likely to be available in the medical record or from the patient or was simple to collect from any source without adding unduly to the healthcare professionals’ workload. Validity and feasibility were each rated on a 9-point scale, where 1 meant definitely not valid or not feasible and 9 definitely valid or feasible. A questionnaire item invited the panellists to comment on each of the indicators and to suggest additional indicators not included in the list (Appendix S1).

### Panel Selection

Our panellists were members of either the Réseau Mère Enfant de la Francophonie (RMEF, an international group of French-speaking university hospitals specialised in perinatology) or of the staff of hospitals in Paris, France and of the French maternity unit network, who volunteered to participate in our quality improvement programme. We selected the panellists from various geographic regions within France plus a few from other francophone countries (Canada, Belgium, Switzerland, and Lebanon), to ensure that they represented a wide array of clinical approaches and practices. The panellists were also selected to represent a broad range of ages and experience levels. They included obstetricians/gynaecologists, midwives, and paediatricians. All panellists gave consent to participation in the study when they replied to the first Delphi round.

### First Round

The first round began in September 2009 and ended in February 2010. An electronic questionnaire was distributed by regular E-mail, and 3 weeks later the print version of the same questionnaire was mailed to each panellist with a stamped addressed return envelope. Non-responders were contacted by E-mail and telephone.

The median was used to measure the central tendency for the ratings. The final disposition of each indicator was based on the median validity rating, median feasibility rating, and agreement among panellists expressed as a percentage.

In the first round, we selected indicators for which a consensus was achieved regarding validity and feasibility, i.e., for which the median score was in the top tertile (7–9) and at least 65% of panel ratings were in the top tertile.

Feasibility was considered a secondary criterion. Therefore, indicators that were considered valid were selected even when agreement regarding feasibility was low.After the first round, the comments made by the panellists and the additional indicators they suggested were used to clarify the wording of the items and to add indicators.

### Second Round

The second round began in March 2010 and ended in November 2010. Each of the panellists who had participated in the first round was sent the second-round questionnaire by mail. These panellists were also given feedback on the results of the first round (their own previous individual ratings of validity and feasibility, median panel rating for validity and feasibility, and frequency distribution of the validity and feasibility ratings).The panellists were then asked to re-rate each indicator based on both their own opinion and the group response to the previous round. Panellists who failed to respond within the first 3 weeks were contacted by E-mail and telephone.

To be included in the final set, indicators had to have median validity and feasibility ratings in the top tertile (7–9) and 75% agreement among panellists that the rating was in the top tertile. [19] Indicators for which these criteria were not met were discussed during a physical meeting.

It should be noted that indicators meeting the validity criterion but not the feasibility criterion were selected also.

### Physical Meeting

According to the modified Delphi procedure, [20], [21] a half-day physical meeting was held in December 2010, in parallel with a working group convened to discuss our quality programme. During the physical meeting, the panellists discussed indicators requiring further consideration and clarified the definitions of the indicators.

The meeting was chaired by three of us (OS, RB and CA). The panel was informed of the median ratings given by all panellists and of the percentage of agreement for validity and feasibility. Indicators with a low rate of agreement in the second round were discussed to identify areas of disagreement.

## Results

Of the 56 experts who were selected to participate in the study, 38 were obstetricians/gynaecologists, 17 were midwives, and 1 was a paediatrician. Among them, 35 (63%) participated in first round (see names in the acknowledgements section). **Table 1** reports the main characteristics of the panellists. Of the obstetricians and midwives, 28 had more than 10 years of experience, including 9 obstetricians who were unit directors or professors and 3 who were head midwives. The 35 participants worked in 20 maternity units, of which 13 were in teaching hospitals.

**Table 1:** Panel characteristics.

Characteristics (N = 35)	
**Sex,** n (%)	
Female	20 (57.1)
Male	15(42.9)
**Age (years),** median (q1, q 3)	44 (37–52)
**Years of experience,** median (q1, q 3)	18 (12–25)
**Practice location,** n (%)	
Teaching hospital	28 (80)
Non-teaching hospital	5 (14.2)
Maternity network	1 (2.9)
Private hospital	1(2.9)
**Job title**, n (%)	
Hospital physician, consultant	12 (34.3)
Midwife	12 (34.3)
Professor	9 (25.7)
Senior Registrar/Clinical Lecturer	2 (5.7)
**Geographical origin (**N participants/N centres)	
France	28/14
Canada	2/2
Belgium	3/2
Switzerland	1/1
Lebanon	1/1

### First round

**Figure 1:**
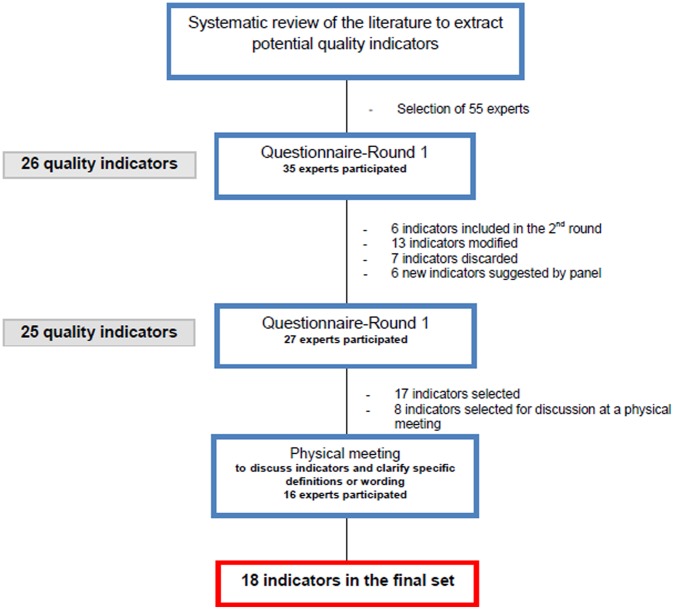
Modified Delphi process used to select and prioritise quality indicators for obstetrical care.

**Figure 1** shows the number of indicators that were included or suggested in each of the two rounds. As shown in **Table 2**, of the 35 first-round participants, more than 65% gave validity ratings in the top tertile (7–9) to 19 of the 26 indicators. Feasibility ratings were higher than validity ratings, with 24 of the 26 indicators having median feasibility values in the top tertile. Epidural analgesia and maternal ICU admission had the validity ratings with the strongest agreement (88.6% and 88.2% respectively). Among indicators included in the second round, 6 were not modified (QI1, QI2, QI10, QI15, QI22, and QI23) and 13 were modified based on the panellists’ comments. For example, for ‘epidural analgesia’, the denominator was changed from ‘total population of women’ to ‘women who gave vaginal birth’.

**Table 2:** Validity and feasibility scores assigned during the first and second Delphi rounds.

	Round 1					Round 2				
	Validity		Feasibility		Status	Validity		Feasibility		Status
Quality indicators	Median	% agreement (7–9)	Median	% agreement (7–9)		Median	% agreement (7–9)	Median	% agreement (7–9)	
**#1**. Caesarean section before labour	8	71.4	9	91.4	included	8	81.5	9	100	Selected
**#2**. Caesarean section during labour	8	80	9	88.2	included	9	81.5	9	100	Selected
**#3**. Maternal ICU admission	8.5	88.2	9	85.7	modified	8.5	80.8	9	92.3	Selected
**#4**. Blood transfusion	8	85.7	9	82.8	modified	8	92.6	9	92.6	Selected
**#5**. Sulprostone use	6	48.4	9	77.4	deleted	/	/	/	/	/
**#6**. Third/fourth-degree perineal tear (full-thickness tears)	8	75.7	9	78.8	modified	9	69.2	9	92.3	To be discussed
**#7**. Examination of uterus	6	45.7	8	77.1	deleted	/	/	/	/	/
**#8**. Epidural analgesia	8	88.6	9	97.1	modified	8	88.9	9	100	Selected
**#9**. Failed tocolytic therapy	5	31.4	6	45.7	deleted	/	/	/	/	/
**#10**. Uterine rupture	9	76.5	9	81.8	included	9	85.2	9	92.6	Selected
**#11**. Intact perineum	8	74.3	9	77.1	modified	8	81.5	9	85.2	Selected
**#12**. Breastfeeding	7	81.1	9	74.2	modified	7	76.9	9	81.5	Selected
**#13**. Vaginal sampling in the 9^th^ month to screenfor Streptococcus group B carriage	8	71.4	5	32	modified	8	88.9	7	55.6	Selected
**#14**. Nosocomial infection of surgical site	9	85.3	7	58.5	modified	9	92.6	8	70.3	Selected
**#15**. Maternal-foetal group B streptococcus infection	8	74.3	8	68.6	included	8	73	8	74.1	To be discussed
**#16**. Nuchal translucency measurement during the first trimester of pregnancy	9	87.5	8.5	75	modified	9	96.3	8	85.2	Selected
**#17**. Three-marker screening offered during the first trimester of pregnancy	8	77.4	7.5	56.7	modified	8	88.9	8	85.2	Selected
**#18**. Spontaneous premature labour	7	68.7	9	75	modified	7	69.2	9	96.2	To be discussed
**#19**. Caesarean section before labour in low-risk woman	9	87.9	8	78.1	modified	9	85.2	8	77.8	Selected
**#20**. Caesarean section during labour in low-risk woman	9	84.8	9	78.8	modified	8	88.5	9	80.8	Selected
**#21**. Brachial plexus palsy	8	77.1	9	80	deleted	/	/	/	/	/
**#22**. Rate of non-low-birth-weight neonatesadmitted to NICU	8	65.7	8	88.6	included	8	81.5	9	96.3	Selected
**#23**. Instrumental vaginal delivery	7	77.1	9	97.7	included	8	85.2	9	100	Selected
**#24**. Birth <32 wk with Apgar <9 at 5 min	5	38.2	9	88.2	deleted	/	/	/	/	/
**#25**. Birth between 32 and 36 wk with Apgar <9 at 5 min	6	47.1	9	94.1	deleted	/	/	/	/	/
**#26**. Birth ≥37 wk with Apgar <9 at 5 min	7	57.1	9	82.8	deleted	/	/	/	/	/
**#27**. Birth between 24 and 32 wk with Apgar <7 at 5 min					added	6	46.2	9	80.8	To be discussed
**#28**. Birth between 33 and 36 wk with Apgar <7 at 5 min					added	8	62.9	9	88.9	To be discussed
**#29**. Birth ≥37 wk with Apgar <7 at 5 min					added	9	77.8	9	92.6	Selected
**#30**. Episiotomy rate					added	7	65.4	9	96.2	To be discussed
**#31**. Labour induction in the absence of abnormalities					added	7	57.5	8	73.1	To be discussed
**#32**. Prostaglandin use					added	7	66.7	9	95.6	To be discussed

From 1 to 26: indicators included in the first round.

From 27 to 32: new indicators added by panellists for evaluation in the second round.

“Status: included” means that the indicator was included in the second round without any change; “status: modified” means the indicator was included in the second round after being modified; “status: deleted” means the indicator was not included in the second round; and “status: added” means the indicator was added for inclusion in the second round on the suggestion of the panellists.

**#21**. Brachial plexus palsy was rated as valid but was not selected after discussion with the researchers and a review of the comments by the panellists. It was considered relatively rare and ill-suited to continuous CUSUM-chart monitoring.

**#24**., **#25**., **#26**. Apgar to 9 at 5 min was considered to high and not relevant for neonatal outcomes.

ICU: intensive care unit; NICU: neonatal intensive care unit; Wk: weeks of amenorrhoea.

Of the remaining 7 indicators, 6 (6/26, 23%) were discarded because they had median validity ratings below 7 (QI 5, QI7, QI9, QI24, and QI25). One indicator, brachial plexus palsy, was rated as valid but was not selected after discussion with the researchers and a review of the comments by the panellists. Brachial plexus palsy was considered relatively rare and ill-suited to continuous CUSUM-chart monitoring. Six new indicators were added (QI27, QI28, QI29, QI30, QI31, and QI32), producing 25 indicators for the second round.

### Second Round

The second round was completed by 27 (27/35: 77%) panellists. **Table 2** shows the results. More than 75% of the panellists gave top-tertile (7–9) validity ratings to 17 indicators. The 8 remaining indicators were discussed during the physical meeting. Nuchal translucency measurement during the first trimester of pregnancy (QI16) had the highest validity ratings (median, 9 with 96.3% of agreement).

Feasibility was rated in the top tertile for all 25 indicators, and the median feasibility rating was 9 for 18 indicators. Of the six indicators added between the two rounds, only ‘birth ≥37 wk with Apgar <7 at 5 min’ was rated with strong agreement.

**Table 3** shows the variations in the total number of indicators given top-tertile validity ratings after the first and second rounds in each group of panellists. Overall, in the first round with 26 indicators, the paediatrician gave top-tertile validity ratings to the smallest number of indicators (10/26, 38%) and midwives to the largest number (25/26, 96%). The same occurred in the second round with top-tertile validity ratings given by paediatricians to the smallest number of indicators (10/25, 40%) and by midwives to the largest number (21/25, 84%).

**Table 3:** Percentage of indicators rated as valid by all groups of panellists.

	Indicators rated valid in the Delphi process n/N (%)	
Group	Delphi round 1*	Delphi round 2†
Obstetricians- gynaecologists	**15/26 (58)**	**(14/25) (56)**
Midwives	**25/26 (96)**	**(21/25) (84)**
Paediatrician	**10/26 (38)**	**(10/25) (40)**

n/N (number of indicators rated valid/total number of indicators assessed ).

* the indicator was considered valid if the median rating was 7 to 9 and at least 65% of panellists gave a rating in the highest tertile (7–9).

† the indicator was considered valid if the median rating was 7 to 9 and at least 75% of panellists gave a rating in the highest tertile (7–9).

### Physical Meeting

During the physical meeting, 16 of the panellists (at least one from each participating maternity centre) and a number of other individuals interested in our quality improvement programme discussed the 8 indicators that were not selected during the second round. Of these 8 indicators, only 1 (QI 6, third/fourth-degree perineal tear) was selected. Of the 5 additional indicators suggested by the panellists at the first round but not selected during the second round (QI27, QI28, QI30, QI31 and QI32), none was selected during the physical meeting.

For several indicators, the panellists decided during the meeting to clarify the definition of the target population (denominator) and/or the language used to describe the indicator. For example, the population of low-risk women was defined as women aged between 18 and 40 years with a singleton pregnancy, cephalic presentation, and no underlying co-morbidities during the pregnancy (e.g., diabetes or hypertension, uterine scarring, admission before 37 weeks’ gestational age…), no aspirin use during pregnancy, and no non-routine investigations such as foetal imaging by magnetic resonance imaging or computed tomography. **Table 4** lists the final 18 indicators selected (10 process and 8 outcome indicators), with the definitions of the numerators and denominators.

**Table 4:** Final list of quality indicators for obstetrical care chosen by the expert panel.

QUALITY INDICATORS	NUMERATOR	DENOMINATOR	Unwanted direction ofrate change
Management of pregnancy and labour			
**#01**. Nuchal translucency measurementduring the first trimester of pregnancy	Number of women with nuchal translucency measurement during the first trimester of pregnancy	Total number of women delivered	decrease
**#02**. Three-marker screening performedduring the first trimester of pregnancy	Number of women with three-marker screeningduring the first trimester	Total number of women delivered	decrease
**#03**. Vaginal sampling in the 9^th^ month to screen for Streptococcus group B carriage	Number of women who underwent vaginalsampling in the 9^th^ month to screen for Streptococcus group B carriage	Total number of women delivered	decrease
**#04**. Epidural analgesia use	Number of women with epidural analgesia use	Total number of women who delivered vaginally	decrease
**#05**. Caesarean section before labour	Number of caesarean sections before labour	Total number of women delivered	Increase
**#06**.Caesarean section during labour	Number of caesarean sections during labour	Total number of women delivered	Increase
**#07**. Third/fourth-degree perineal tear(full-thickness tears)	Number of women with third/fourth-degree perineal tears	Total number of women who delivered vaginally	Increase
**#08**. Uterine rupture	Number of women with uterine rupture	Total number of women delivered	Increase
**#09**. Intact perineum	Number of women with intact perineum	Total number of women who delivered vaginally	decrease
**#10**. Nosocomial infection of surgical site	Number of women with nosocomial infections	Number of women who had surgery	Increase
**#11**. Blood transfusion during and/or after delivery	Number of women given blood transfusions during and/or after delivery (delivery related blood loss >1500 mL)	Total number of women delivered	Increase
**#12**. Maternal ICU transfer and/or admission	Number of women transferred and/or admitted to the ICU	Total number of women delivered	Increase
**#13**. Decision to breastfeed at discharge	Number of women who decided to breastfeed at discharge	Total number of women dischargedhome with a live baby	decrease
**Management of low risk women**			
**#14**. Caesarean section before labour inlow-risk woman	Number of caesarean sections before labour inlow-risk women	Women aged between 18 and 40 years with a singleton pregnancy, cephalic presentation, and no underlying co-morbidities during the pregnancy (e.g., diabetes or hypertension, uterine scarring, admission before 37 weeks’ gestational age…), no aspirin use during pregnancy, and no non-routine investigations such as foetal imaging by magnetic resonance imaging or computed tomography.	Increase
**#15**. Caesarean section during labour in low-risk woman	Number of caesarean sections during labour in low-risk women	Same definition as above	Increase
**Management of new born**			
**#16**. Instrumental vaginal delivery	Number of neonates delivered by instrumental extraction (using obstetric forceps or vacuum extractor)	Total number of vaginally births (all live born neonates including those with birth defects	Increase
**#17**. Rate of non-low-birth-weight neonates admitted to the NICU	Number of NICU admissions of neonates >2500 g without birth defects	Total number of neonates (all live born neonates including those with birth defects	Increase
**#18**. Birth ≥37 wk with Apgar <7 at 5 min	Number of births ≥37 wk with Apgar <7 at 5 min	Total number of births 37 wk	Increase

ICU: intensive care unit; NICU: Neonatal intensive care unit; Wk: weeks of amenorrhoea.

## Discussion

We identified 18 quality indicators designed to assess the overall quality of obstetrical care and to be routinely monitored in maternity units. This list resulted from a consensus among 35 multidisciplinary experts who varied widely in terms of years of experience and clinical practice. The final set of 18 indicators is a starting point for developing a vast obstetrical-care quality-improvement programme based on the CUSUM chart method. [14] CUSUM charts are obtained by computing the cumulative difference between each observed value of the quality indicator at a specific moment and a target value representing rates defined as acceptable or not acceptable, taking into account the information available for all previous points. The selection of quality indicators is an important preliminary step to the establishment of CUSUM charts. Quality indicators should reflect critical elements of the process of care that is being evaluated. Furthermore, they should be easy to assess based on routinely collected data.

Previous studies have sought to identify quality indicators for obstetrical care. However, to the best of our knowledge, this is the first study to use the modified Delphi technique. Indeed, numerous quality measures have been suggested, but monitoring all of them would be impractical. [22] In obstetrics, outcomes are often used as performance measures. Draycott and colleagues [23] noted that 290 maternity outcomes in 96 clinical categories had been selected by four professional bodies (the Royal College of Obstetricians & Gynaecologists [RCOG], American College of Obstetricians & Gynecologists [ACOG], Royal Australian and New Zealand College of Obstetricians & Gynaecologists [RANZCOG], and Society of Obstetricians and Gynaecologists of Canada [SOGC]). Other initiatives such as the Quality Improvement for Emergency Obstetric Care toolbook have been developed but are suitable only for emergency care [24].

Waterstone et al. [25] described outcomes including maternal haemorrhage, severe preeclampsia, eclampsia, and sepsis and found that 1.2% of patients in 19 maternity units experienced one of these events. These events clearly reflect the quality of obstetrical care and deserve to be recorded. However, some of them are not well suited to continuous monitoring using a dashboard. Indeed, most obstetrical patients are healthy women who receive relatively straightforward care, and advances in treatments and technologies have made these events relatively rare. In addition, the occurrence of only one such event would trigger an investigation. For example, given that maternal death in industrialised countries is rare and often preventable by good healthcare, [26] maternal mortality lacks sensitivity as a marker for the quality of obstetrical care. Nevertheless, sentinel event analysis of maternal deaths should be a critical component of local efforts to improve quality assurance and the quality of care. [27] Another example is the rate of brachial plexus palsy. Despite the strong consensus in the first round of our study, this indicator was not selected, because the occurrence of a single case would lead to an audit or to an evaluation during mortality-morbidity reviews. Nowadays, parents expect not only survival of the mother and neonate, but also maintenance of good health, optimal comfort for the mother and baby, and an overall positive experience. Therefore, other quality indicators must be used. Evaluations of obstetrical care now usually rely on the rates of events such as caesarean section and nosocomial infection. These indicators reflect important elements of the process of care and are widely used in national surveys and in the benchmarking of on-going care within and among healthcare institutions [22] [28].

Therefore, identification of the most useful indicators remains a priority to improve the quality of obstetrical care. Differences in opinions across healthcare professionals complicate the identification of indicators. However, the use of structured methods such as the Delphi process helps to develop a consensus.

Although the Delphi process is a well-validated method for assessing opinions, [7] several methodological issues require discussion. First, the definition of a ‘consensus’ among participants is not agreed on, and various definitions were used in previous studies. [29] We required a higher agreement rate in the second than in the first round, as second-round participants were aware of the survey contents and received feedback about the first round, including the median ratings with the ranges, the participants’ responses, and a summary of all the comments received. These data allowed each participant to assess his or her position relative to the rest of the group, which may have influenced the response to the second round.

Second, the Delphi process requires a multidisciplinary panel of experts to enhance the credibility and acceptance of the final set of indicators and to ensure that all aspects of quality of care are discussed. [6] The panel should reflect the full range of stakeholders. Our panel included at least one member of the three main professions involved in obstetrical care. We found differences in ratings across these three professions, indicating differences in viewpoints about quality of care, [30], [31] which enriched the results of the Delphi procedure. Although most of the panellists worked in France, they reflected a diversity of opinions and practices, since they worked in 20 different maternity units that varied in terms of case-mix and obstetrical practice patterns. Such heterogeneity in practice patterns may improve the performance of decision-making groups [32] and help to increase the content validity of the results.

Third, as we addressed quality of care from the perspective of healthcare professionals, we did not include patients in our panel. Patients’ viewpoints are important to consider when seeking to improve healthcare [33]. We will measure patient satisfaction in a second step of our research programme, using appropriate tools such as satisfaction questionnaires to collect patients’ opinions about quality of care [34].

Fourth, Delphi participants are typically polled individually, using self-administered questionnaires in most cases, with no physical meeting. The procedure generally involves two or more rounds, with feedback of the results of each round to the group before the next round. The absence of a physical meeting prevents one or a few experts from dominating the consensus process. However, a physical meeting may help the participants to identify reasons for disagreements. [35] This modification to the Delphi procedure is widely used: more than half the Delphi-based studies on quality indicator selection involved one or more physical meetings. [29] In our study, a physical meeting was held to resolve uncertainties and to clarify the language describing the quality indicators. For example, the term ‘low-risk women’ was deemed insufficiently specific and a more detailed definition was therefore developed during the meeting. This would not have been possible using only the participants’ feedback after each Delphi round.Finally, although some of the panellists failed to participate in all the steps of the Delphi survey (suggesting some degree of weariness with the process), the participation rate was within the range of previous studies [29] and incomplete participation does not undermine the validity of the results.

The Delphi process allowed us to identify two groups of indicators conforming to the traditional Donabedian approach to quality assessment, namely process and outcome indicators.Structure indicators, which are fairly well controlled during accreditation processes, were not mentioned in our study. The selected indicators were relevant to various populations, i.e., all pregnant women, low-risk pregnant women and all neonates.

The selected indicators concerning the management of all pregnant women are currently the most widely accepted indicators. An example is the caesarean section rate, which is clearly defined, easily collectible, and relevant to efforts aimed at decreasing maternal morbidity and healthcare costs. Both caesarean section during labour and caesarean section before labour were selected, given the different levels of risk, with caesarean section during labour being associated with higher maternal mortality and morbidity rates. Caesarean section rates were determined both in the overall population of women and in low-risk women. In the overall population, caesarean section rates depend chiefly on the case-mix in the maternity centre and may consequently vary across centres. In low-risk women, very low adverse event rates are expected and the caesarean section rates therefore show little variation across centres, allowing comparisons of different centres.

Other indicators reflecting severe morbidity related to pregnancy and labour were selected, such as uterine rupture, maternal ICU transfer and/or admission, and third/fourth-degree perineal tear. In addition to this last indicator, representing a severe and rare event, the panel selected ‘intact perineum’ to reflect not only absence of third/fourth-degree tears, but also absence of less severe tears during instrumental vaginal delivery and absence of episiotomy. An intact perineum is an important goal to maximise patient comfort, minimise pain, and ensure the absence of residual discomfort due to scarring.

Process indicators were also selected, including epidural analgesia (for pain management), vaginal sampling in the ninth month to screen for streptococcus group B carriage (international recommendation to decrease sepsis), and a decision to breastfeed at discharge. Breastfeeding confers numerous short- and long-term health benefits for the infant. For instance, in healthy full-term babies, breastfeeding has been associated with neurodevelopmental advantages [36], lower rates of obesity [37], and a lower incidence of atopic disorders [38]. However, routine recording of data on breastfeeding may be difficult to achieve, as some mothers do not return to the maternity centre after delivery. A decision by the mother to breastfeed, taken at discharge from the maternity ward, seems to be a reasonable surrogate for actual breastfeeding after discharge. Collecting the decision to breastfeed at discharge is easy and provides an opportunity for the maternity centre staff to provide the mother with support, as well as with breastfeeding information and advice.

Two indicators were related to Down syndrome screening, nuchal translucency on the sonogram and serum marker assays, both assessed during the first trimester. In France, prenatal Down syndrome screening follows guidelines established by the French National Health Authority based on efficacy and safety data, cost considerations, acceptability, and patient preferences [39]. According to these guidelines, nuchal translucency should be assessed on the first-trimester sonogram performed routinely between 11 weeks and 13 weeks 6 days after the last menstrual period and serum markers (pregnancy-associated plasma protein A and free βhCG) should be measured during the same visit.

For neonates, the rate of non-low-birth-weight neonates admitted to the NICU and the instrumental vaginal delivery rate were selected. A decrease in the instrumental vaginal delivery rate is widely accepted as indicating an improvement in the quality of care. Instrumental vaginal delivery is associated with several neonatal adverse effects such as extra- and intracranial haemorrhage [40] and cephalhaematoma [41]. Moreover, instrumental vaginal delivery significantly increases the risk of third or fourth degree perineal tears and of vaginal and cervical lacerations compared with spontaneous vaginal delivery.

Our panellists did not agree about the validity of an Apgar score >9 after 5 minutes (QIs 24, 25, and 26). In the first round, the cut-off was set at 9 to ensure the identification of all potential emergencies including neonatal hypoxia. However, the participants felt that this Apgar cut-off was too high and was not relevant to neonatal outcomes. Consequently, it was brought down to 7 for the second round (QI 27, 28, and 29). The panellists agreed that an Apgar score >7 after 5 minutes was a valid indicator in neonates born at or after 37 weeks’ gestational age (QI 29). This indicator was not considered relevant for babies born before 32 weeks’ gestational age, in keeping with studies showing that immaturity may lead to a low Apgar score in preterm neonates who are relatively healthy. [42], [43] Another study showed centre-to-centre variability in Apgar score assignment in premature infants [44].

A number of process indicators failed to meet our selection criteria. Examples include sulprostone use (IQ5) and prostaglandin use (IQ32). This result is partly due to the heterogeneity of maternity unit practices and of types of healthcare professionals on the panel. For a number of treatments and techniques, substantial disagreement exists regarding the optimal course of action. However, practice uniformity is improving, most notably via the development of standards by international federations (FIGO/UPIGO).

In recent years, maternity units have increasingly adopted computerised data collection systems, allowing the recording of large amounts of data and greatly improving the feasibility of indicators, defined as the ready availability of the data required for indicator assessment. Feasibility exerts a major influence on acceptance and implementation of quality indicators. For example, it has been shown that the validity and feasibility of a specific guideline predicted implementation of the guideline in the clinical setting. [45] The 18 quality indicators selected in our study were all rated as feasible, with median scores between 7 and 9. However, confirmation of their feasibility in everyday practice is needed. The data must be shown to be either routinely available in the medical records or easy to obtain via surveys and interviews.

Data collection using electronic medical record systems has been shown to improve healthcare and to help assess the effectiveness of healthcare practices. [46] All maternity units should consider using a dashboard to plan and improve their services. Dashboard monitoring provides continuous information on clinical performance and governance in everyday practice. This information may help to identify patient safety issues in advance, so that timely and appropriate action can be instituted to ensure high-quality and safe maternity care. Continuous monitoring tools such as the CUSUM chart can be used to monitor a broad range of indicators and to rapidly detect unwanted changes in quality indicator rates in obstetrical practice. [14] Another method, the quality incident notification system, proved able to capture a relatively low rate of obstetrical adverse events in obstetrical care, about half of which were avoidable. [47] This system could be widely used for quality improvement initiatives in obstetric care.

### Conclusion

In conclusion, our study identified a list of 18 quality indicators suitable for routine monitoring. Whether monitoring these indicators would have a clinical impact on maternal and neonatal health remains to be investigated. The final set of 18 indicators represents a starting point for developing a quality improvement programme in French maternity units based on the CUSUM chart method. However, the feasibility of these indicators needs to be assessed by studies conducted under the conditions of everyday practice. In addition, the final set of indicators may require additional clarification to ensure suitability for use in quality improvement strategies. In addition, targets must be defined for each indicator. Ideally, these targets would reflect universally accepted standards. Unfortunately, such standards are rarely available and, consequently, locally accepted standards adapted to the type of population (case-mix), type of healthcare provided, and human and material resources may need to be used instead.

## Supporting Information

Appendix S1 **First Round Delphi Questionnaire.**
(DOC)
