# Supplementary material for: Quality Indicators for Continuous Monitoring to Improve Maternal and Infant Health in Maternity Departments: A Modified Delphi Survey of an International Multidisciplinary Panel
Source: PLoS One. 2013 Apr 5;8(4):e60663. doi: 10.1371/journal.pone.0060663 (PMC3618223; doi:10.1371/journal.pone.0060663)
Supplement: Appendix S1 — First Round Delphi Questionnaire. (DOC) [file pone.0060663.s001.doc]

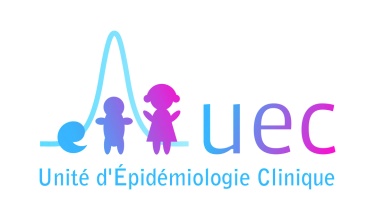


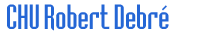

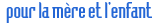


**Clinical Epidemiology Unit**

**Robert Debré Hospital**

**Selection of Quality Indicators in Obstetrical Care**

**DELPHI questionnaire (1^st^ Round )**

In recent years, statistical methods to monitor and control processes, known as Statistical Process Control (SPC), have shown considerable promise for monitoring quality indicators. SPC uses rigorous time-series analysis methods, whose results are reported as graphs of changes in outcome rates over time. Moreover, SPC can help to determine whether these changes are real (i.e., related to a causative factor) or merely a manifestation of natural variability. Among SPC tools, CUmulative SUM (CUSUM) charts are widely used for industrial quality control^6^ and have been found effective for measuring and monitoring healthcare outcomes. In obstetrics, CUSUM charts have been used to monitor Apgar scores or the performance of sonographers in assessing nuchal translucency in the first trimester of pregnancy.

This Delphi survey was the first step in a quality improvement project under way in several maternity units in France. The working hypothesis is that continuous quality-indicator monitoring using CUSUM control charts will increase awareness of quality issues among maternity-unit healthcare workers and permit the rapid detection of small dips in performance, thereby enabling prompt investigations and corrective measures when necessary.

Our aim was to produce relevant and valid indicators that could be routinely monitored in maternity units to guide the development of quality improvement programs.

This questionnaire is the first of two Delphi rounds. During this first round, your task consists in rating a set of quality indicators regarding two dimensions: validity and feasibility.

**Definition of validity**: the extent to which the characteristics of the indicator are appropriate for the concept being assessed

**Definition of feasibility**: an indicator is considered feasible if the information needed to assess it is probably available in the medical record or from the patient or is simple to collect from any source without adding unduly to the healthcare professionals’ workload.

**Rating scale**: The validity and the feasibility of each quality indicator are rated on scales of 1 to 9 points. “1” represents the lowest and “9” the highest rating.

You can make comments on the indicators using the “comment box”. You can also add indicators you feel deserve evaluation in the next round.

To maximise the reliability and consistency of your work, please try to complete the questionnaire in one sitting.

ÉcouterLire phonétiquementDictionnaire

1. **Quality indicators related to the management of the overall population of pregnant women**

(i.e., Total number of women giving birth except those with foetal death in utero or pregnancy termination for medical reasons).

**†Definition of validity**: the extent to which the characteristics of the indicator are appropriate for the concept being assessed, with the indicator being considered a good measure of quality of care. For valid indicators, routine monitoring seems important.

1= you feel this indicator is not important for measuring quality of care / 9 = you feel this indicator is very important for measuring quality of care

**‡ Definition of feasibility**: an indicator is considered feasible if the information needed to assess it is probably available in the medical record or from the patient or is simple to collect from any source without adding unduly to the healthcare professionals’ workload.

1=it is difficult or impossible to find the information needed to monitor the quality indicator/ 9 = it is very easy to find information needed to monitor the quality indicator

| N | Quality indicator | Numerator | Denominator | Validity† | Feasibility‡ |
| --- | --- | --- | --- | --- | --- |
|  | Caesarean section before labour | Number of caesarean sections before labour | Total number of women delivered | | 1 | 2 | 3 | 4 | 5 | 6 | 7 | 8 | 9 | | --- | --- | --- | --- | --- | --- | --- | --- | --- | |  |  |  |  |  |  |  |  |  | | | 1 | 2 | 3 | 4 | 5 | 6 | 7 | 8 | 9 | | --- | --- | --- | --- | --- | --- | --- | --- | --- | |  |  |  |  |  |  |  |  |  | |
|  | Unscheduled caesarean section | Number of unscheduled caesarean sections | Total number of women delivered | | 1 | 2 | 3 | 4 | 5 | 6 | 7 | 8 | 9 | | --- | --- | --- | --- | --- | --- | --- | --- | --- | |  |  |  |  |  |  |  |  |  | | | 1 | 2 | 3 | 4 | 5 | 6 | 7 | 8 | 9 | | --- | --- | --- | --- | --- | --- | --- | --- | --- | |  |  |  |  |  |  |  |  |  | |
|  | Maternal ICU admission | Number of women admitted to the ICU | Total number of women delivered | | 1 | 2 | 3 | 4 | 5 | 6 | 7 | 8 | 9 | | --- | --- | --- | --- | --- | --- | --- | --- | --- | |  |  |  |  |  |  |  |  |  | | | 1 | 2 | 3 | 4 | 5 | 6 | 7 | 8 | 9 | | --- | --- | --- | --- | --- | --- | --- | --- | --- | |  |  |  |  |  |  |  |  |  | |
|  | Blood transfusion | Number of transfused women | Total number of women delivered | | 1 | 2 | 3 | 4 | 5 | 6 | 7 | 8 | 9 | | --- | --- | --- | --- | --- | --- | --- | --- | --- | |  |  |  |  |  |  |  |  |  | | | 1 | 2 | 3 | 4 | 5 | 6 | 7 | 8 | 9 | | --- | --- | --- | --- | --- | --- | --- | --- | --- | |  |  |  |  |  |  |  |  |  | |
|  | Sulprostone use | Number of women given sulprostone | Total number of women | | 1 | 2 | 3 | 4 | 5 | 6 | 7 | 8 | 9 | | --- | --- | --- | --- | --- | --- | --- | --- | --- | |  |  |  |  |  |  |  |  |  | | | 1 | 2 | 3 | 4 | 5 | 6 | 7 | 8 | 9 | | --- | --- | --- | --- | --- | --- | --- | --- | --- | |  |  |  |  |  |  |  |  |  | |
|  | Third/fourth-degree perineal tear (full-thickness tears) | Number of women with third/fourth-degree tears | Total number of women | | 1 | 2 | 3 | 4 | 5 | 6 | 7 | 8 | 9 | | --- | --- | --- | --- | --- | --- | --- | --- | --- | |  |  |  |  |  |  |  |  |  | | | 1 | 2 | 3 | 4 | 5 | 6 | 7 | 8 | 9 | | --- | --- | --- | --- | --- | --- | --- | --- | --- | |  |  |  |  |  |  |  |  |  | |
|  | Examination of uterus | Number of women with examination of uterus | Total number of women | | 1 | 2 | 3 | 4 | 5 | 6 | 7 | 8 | 9 | | --- | --- | --- | --- | --- | --- | --- | --- | --- | |  |  |  |  |  |  |  |  |  | | | 1 | 2 | 3 | 4 | 5 | 6 | 7 | 8 | 9 | | --- | --- | --- | --- | --- | --- | --- | --- | --- | |  |  |  |  |  |  |  |  |  | |
|  | Epidural analgesia | Number of women with epidural analgesia | Total number of women | | 1 | 2 | 3 | 4 | 5 | 6 | 7 | 8 | 9 | | --- | --- | --- | --- | --- | --- | --- | --- | --- | |  |  |  |  |  |  |  |  |  | | | 1 | 2 | 3 | 4 | 5 | 6 | 7 | 8 | 9 | | --- | --- | --- | --- | --- | --- | --- | --- | --- | |  |  |  |  |  |  |  |  |  | |
|  | Failed tocolytic therapy | Number of women with failed tocolytic therapy | Total number of women | | 1 | 2 | 3 | 4 | 5 | 6 | 7 | 8 | 9 | | --- | --- | --- | --- | --- | --- | --- | --- | --- | |  |  |  |  |  |  |  |  |  | | | 1 | 2 | 3 | 4 | 5 | 6 | 7 | 8 | 9 | | --- | --- | --- | --- | --- | --- | --- | --- | --- | |  |  |  |  |  |  |  |  |  | |
|  | Uterine rupture | Number of women with uterine rupture | Total number of women | | 1 | 2 | 3 | 4 | 5 | 6 | 7 | 8 | 9 | | --- | --- | --- | --- | --- | --- | --- | --- | --- | |  |  |  |  |  |  |  |  |  | | | 1 | 2 | 3 | 4 | 5 | 6 | 7 | 8 | 9 | | --- | --- | --- | --- | --- | --- | --- | --- | --- | |  |  |  |  |  |  |  |  |  | |
|  | Intact perineum | Number of women with intact perineum | Total number of women | | 1 | 2 | 3 | 4 | 5 | 6 | 7 | 8 | 9 | | --- | --- | --- | --- | --- | --- | --- | --- | --- | |  |  |  |  |  |  |  |  |  | | | 1 | 2 | 3 | 4 | 5 | 6 | 7 | 8 | 9 | | --- | --- | --- | --- | --- | --- | --- | --- | --- | |  |  |  |  |  |  |  |  |  | |
|  | Breastfeeding | Number of women who breastfeed at discharge | Total number of women | | 1 | 2 | 3 | 4 | 5 | 6 | 7 | 8 | 9 | | --- | --- | --- | --- | --- | --- | --- | --- | --- | |  |  |  |  |  |  |  |  |  | | | 1 | 2 | 3 | 4 | 5 | 6 | 7 | 8 | 9 | | --- | --- | --- | --- | --- | --- | --- | --- | --- | |  |  |  |  |  |  |  |  |  | |
|  | Vaginal sampling in the 9^th^ month to screen for Streptococcus group B carriage | Number of women who underwent vaginal sampling in the 9^th^ month to screen for Streptococcus group B carriage | Total number of women | | 1 | 2 | 3 | 4 | 5 | 6 | 7 | 8 | 9 | | --- | --- | --- | --- | --- | --- | --- | --- | --- | |  |  |  |  |  |  |  |  |  | | | 1 | 2 | 3 | 4 | 5 | 6 | 7 | 8 | 9 | | --- | --- | --- | --- | --- | --- | --- | --- | --- | |  |  |  |  |  |  |  |  |  | |
|  | Nosocomial infection of surgical site | Number of women with nosocomial infection of surgical site | Total number of women | | 1 | 2 | 3 | 4 | 5 | 6 | 7 | 8 | 9 | | --- | --- | --- | --- | --- | --- | --- | --- | --- | |  |  |  |  |  |  |  |  |  | | | 1 | 2 | 3 | 4 | 5 | 6 | 7 | 8 | 9 | | --- | --- | --- | --- | --- | --- | --- | --- | --- | |  |  |  |  |  |  |  |  |  | |
|  | Maternal-foetal group B streptococcus infection | Number of maternal-foetal group B streptococcus infections | Total number of women | | 1 | 2 | 3 | 4 | 5 | 6 | 7 | 8 | 9 | | --- | --- | --- | --- | --- | --- | --- | --- | --- | |  |  |  |  |  |  |  |  |  | | | 1 | 2 | 3 | 4 | 5 | 6 | 7 | 8 | 9 | | --- | --- | --- | --- | --- | --- | --- | --- | --- | |  |  |  |  |  |  |  |  |  | |

**Comments**…………………………………………………………………………………………………………………………………………………………………………………………………………………………………………………………………………………………………………………………………………………………………………………………………………………………………………………………………………………………………………………………………………………………………………………………………………………………………………

**Additional quality indicators** ………………………………………………………………………………………………………………………………………………………………………………………………………………………………………………………………………………………………………………………………………………………………………………………………………………………………………………………………………………………………………………………………………………………………………………………………………………………………………….

1. **Quality indicators related to the management of women followed from the first trimester of pregnancy**

**†Definition of validity**: the extent to which the characteristics of the indicator are appropriate for the concept being assessed, with the indicator being considered a good measure of quality of care. For valid indicators, routine monitoring seems important.

1= you feel this indicator is not important for measuring quality of care / 9 = you feel this indicator is very important for measuring quality of care

**‡ Definition of feasibility**: an indicator is considered feasible if the information needed to assess it is probably available in the medical record or from the patient or is simple to collect from any source without adding unduly to the healthcare professionals’ workload.

1=it is difficult or impossible to find the information needed to monitor the quality indicator/ 9 = it is very easy to find information needed to monitor the quality indicator

| N | Quality indicator | Numerator | Denominator | Validity† | Feasibility‡ |
| --- | --- | --- | --- | --- | --- |
|  | Nuchal translucency measurement during the first trimester of pregnancy | Number of women with nuchal translucency measurements during the first trimester of pregnancy | Total number of women followed from the first trimester of pregnancy | | 1 | 2 | 3 | 4 | 5 | 6 | 7 | 8 | 9 | | --- | --- | --- | --- | --- | --- | --- | --- | --- | |  |  |  |  |  |  |  |  |  | | | 1 | 2 | 3 | 4 | 5 | 6 | 7 | 8 | 9 | | --- | --- | --- | --- | --- | --- | --- | --- | --- | |  |  |  |  |  |  |  |  |  | |
|  | Three-marker screening offered during the first trimester of pregnancy | Number of women with three-marker screening during the first trimester of pregnancy | Total number of women followed from the first trimester of pregnancy | | 1 | 2 | 3 | 4 | 5 | 6 | 7 | 8 | 9 | | --- | --- | --- | --- | --- | --- | --- | --- | --- | |  |  |  |  |  |  |  |  |  | | | 1 | 2 | 3 | 4 | 5 | 6 | 7 | 8 | 9 | | --- | --- | --- | --- | --- | --- | --- | --- | --- | |  |  |  |  |  |  |  |  |  | |
|  | Spontaneous premature labour | Number of women with spontaneous premature labour | Total number of women followed from the first trimester of pregnancy | | 1 | 2 | 3 | 4 | 5 | 6 | 7 | 8 | 9 | | --- | --- | --- | --- | --- | --- | --- | --- | --- | |  |  |  |  |  |  |  |  |  | | | 1 | 2 | 3 | 4 | 5 | 6 | 7 | 8 | 9 | | --- | --- | --- | --- | --- | --- | --- | --- | --- | |  |  |  |  |  |  |  |  |  | |

**Comments**…………………………………………………………………………………………………………………………………………………………………………………………………………………………………………………………………………………………………………………………………………………………………………………………………………………………………………………………………………………………………………………………………………………………………………………………………………………………………………

**Additional quality indicators** ………………………………………………………………………………………………………………………………………………………………………………………………………………………………………………………………………………………………………………………………………………………………………………………………………………………………………………………………………………………………………………………………………………………………………………………………………………………………………….

1. **Quality indicators related to the management of low-risk pregnant women**

**†Definition of validity**: the extent to which the characteristics of the indicator are appropriate for the concept being assessed, with the indicator being considered a good measure of quality of care. For valid indicators, routine monitoring seems important.

1= you feel this indicator is not important for measuring quality of care / 9 = you feel this indicator is very important for measuring quality of care

**‡ Definition of feasibility**: an indicator is considered feasible if the information needed to assess it is probably available in the medical record or from the patient or is simple to collect from any source without adding unduly to the healthcare professionals’ workload.

1=it is difficult or impossible to find the information needed to monitor the quality indicator/ 9 = it is very easy to find information needed to monitor the quality indicator

| N | Quality indicator | Numerator | Denominator | Validity† | Feasibility‡ |
| --- | --- | --- | --- | --- | --- |
|  | Scheduled caesarean section in low-risk woman | Number of scheduled caesarean sections in low-risk women | Total number of low-risk women | | 1 | 2 | 3 | 4 | 5 | 6 | 7 | 8 | 9 | | --- | --- | --- | --- | --- | --- | --- | --- | --- | |  |  |  |  |  |  |  |  |  | | | 1 | 2 | 3 | 4 | 5 | 6 | 7 | 8 | 9 | | --- | --- | --- | --- | --- | --- | --- | --- | --- | |  |  |  |  |  |  |  |  |  | |
|  | Unscheduled caesarean section in low-risk woman | Number of unscheduled caesarean sections in low-risk women | Total number of low-risk women | | 1 | 2 | 3 | 4 | 5 | 6 | 7 | 8 | 9 | | --- | --- | --- | --- | --- | --- | --- | --- | --- | |  |  |  |  |  |  |  |  |  | | | 1 | 2 | 3 | 4 | 5 | 6 | 7 | 8 | 9 | | --- | --- | --- | --- | --- | --- | --- | --- | --- | |  |  |  |  |  |  |  |  |  | |

**Comments**…………………………………………………………………………………………………………………………………………………………………………………………………………………………………………………………………………………………………………………………………………………………………………………………………………………………………………………………………………………………………………………………………………………………………………………………………………………………………………

**Additional quality indicators** ………………………………………………………………………………………………………………………………………………………………………………………………………………………………………………………………………………………………………………………………………………………………………………………………………………………………………………………………………………………………………………………………………………………………………………………………………………………………………….

1. **Quality indicators related to the management of neonates**

**†Definition of validity**: the extent to which the characteristics of the indicator are appropriate for the concept being assessed, with the indicator being considered a good measure of quality of care. For valid indicators, routine monitoring seems important.

1= you feel this indicator is not important for measuring quality of care / 9 = you feel this indicator is very important for measuring quality of care

**‡ Definition of feasibility**: an indicator is considered feasible if the information needed to assess it is probably available in the medical record or from the patient or is simple to collect from any source without adding unduly to the healthcare professionals’ workload.

1=it is difficult or impossible to find the information needed to monitor the quality indicator/ 9 = it is very easy to find information needed to monitor the quality indicator

| N | Quality indicator | Numerator | Denominator | Validity† | Feasibility‡ |
| --- | --- | --- | --- | --- | --- |
|  | Brachial plexus injury | Number of neonates with brachial plexus injury | Total number of neonates | | 1 | 2 | 3 | 4 | 5 | 6 | 7 | 8 | 9 | | --- | --- | --- | --- | --- | --- | --- | --- | --- | |  |  |  |  |  |  |  |  |  | | | 1 | 2 | 3 | 4 | 5 | 6 | 7 | 8 | 9 | | --- | --- | --- | --- | --- | --- | --- | --- | --- | |  |  |  |  |  |  |  |  |  | |
|  | NICU admission of neonates >2.500 g without birth defects | Number of NICU admissions of neonates >2500 g without birth defects | Total number of neonates | | 1 | 2 | 3 | 4 | 5 | 6 | 7 | 8 | 9 | | --- | --- | --- | --- | --- | --- | --- | --- | --- | |  |  |  |  |  |  |  |  |  | | | 1 | 2 | 3 | 4 | 5 | 6 | 7 | 8 | 9 | | --- | --- | --- | --- | --- | --- | --- | --- | --- | |  |  |  |  |  |  |  |  |  | |
|  | Instrumental extraction | Number of neonates delivered by instrumental extraction | Total number of neonates | | 1 | 2 | 3 | 4 | 5 | 6 | 7 | 8 | 9 | | --- | --- | --- | --- | --- | --- | --- | --- | --- | |  |  |  |  |  |  |  |  |  | | | 1 | 2 | 3 | 4 | 5 | 6 | 7 | 8 | 9 | | --- | --- | --- | --- | --- | --- | --- | --- | --- | |  |  |  |  |  |  |  |  |  | |
|  | Birth <32 wk with Apgar <9 at 5 min | Number of births <32 wk with Apgar <9 at 5 min | Total number of births <32 wk | | 1 | 2 | 3 | 4 | 5 | 6 | 7 | 8 | 9 | | --- | --- | --- | --- | --- | --- | --- | --- | --- | |  |  |  |  |  |  |  |  |  | | | 1 | 2 | 3 | 4 | 5 | 6 | 7 | 8 | 9 | | --- | --- | --- | --- | --- | --- | --- | --- | --- | |  |  |  |  |  |  |  |  |  | |
|  | Birth between 32 and 36 wk with Apgar <9 at 5 min | Number of births between 32 and 36 wk with Apgar <9 at 5 min | Total number of births between 32 and 36 wk | | 1 | 2 | 3 | 4 | 5 | 6 | 7 | 8 | 9 | | --- | --- | --- | --- | --- | --- | --- | --- | --- | |  |  |  |  |  |  |  |  |  | | | 1 | 2 | 3 | 4 | 5 | 6 | 7 | 8 | 9 | | --- | --- | --- | --- | --- | --- | --- | --- | --- | |  |  |  |  |  |  |  |  |  | |
|  | Birth ≥37 wk with Apgar <9 at 5 min | Number of births ≥ 37 wk with Apgar <9 at 5 min | Total number of births ≥37 wk | | 1 | 2 | 3 | 4 | 5 | 6 | 7 | 8 | 9 | | --- | --- | --- | --- | --- | --- | --- | --- | --- | |  |  |  |  |  |  |  |  |  | | | 1 | 2 | 3 | 4 | 5 | 6 | 7 | 8 | 9 | | --- | --- | --- | --- | --- | --- | --- | --- | --- | |  |  |  |  |  |  |  |  |  | |

**Comments**…………………………………………………………………………………………………………………………………………………………………………………………………………………………………………………………………………………………………………………………………………………………………………………………………………………………………………………………………………………………………………………………………………………………………………………………………………………………………………

**Additional quality indicators** ………………………………………………………………………………………………………………………………………………………………………………………………………………………………………………………………………………………………………………………………………………………………………………………………………………………………………………………………………………………………………………………………………………………………………………………………………………………………………….
